# Antioxidant, antimicrobial and antiproliferative activities of *Anthemis palestina* essential oil

**DOI:** 10.1186/1472-6882-14-297

**Published:** 2014-08-12

**Authors:** Sanaa K Bardaweel, Khaled A Tawaha, Mohammad M Hudaib

**Affiliations:** Department of Pharmaceutical Sciences, Faculty of Pharmacy, The University of Jordan, Queen Rania Street, Amman, 11942 Jordan

**Keywords:** *Anthemis palestina*, Essential (volatile) oil, Antioxidant, Antimicrobial, Antiproliferative

## Abstract

**Background:**

*Anthemis palestina* (Asteraceae) extends across the Mediterranean region, southwest Asia and eastern Africa. Although traditionally used for several applications, *in vitro* investigation of biological functions associated with *Anthemis palestina* essential oil had never been reported.

**Methods:**

The air-dried flowers of *Anthemis palestina* were subjected to hydrodistillation to yield the oil. The antioxidant activity of the hydrodistilled oil was characterized using various *in vitro* model systems such as DPPH, ferric-reducing antioxidant power and hydroxyl radical scavenging activity. Antibacterial activity was tested against six bacterial species, representing both Gram positive and Gram negative bacteria. Antifungal activity was evaluated using three *Candida* species. The minimum inhibitory concentration (MIC) for each examined microorganism was determined using the microdilution method. The oil’s antiproliferative effects against eight human cancer cell lines were also studied and the lethal doses that resulted in 50% reduction of cell viability (LD_50_) were determined.

**Results:**

The results indicate that the essential oil of *Anthemis palestina* exhibited substantial antioxidant activities as demonstrated with DPPH, ferric reducing antioxidant power, and hydroxyl radical scavenging activity. In addition, a broad-spectrum antibacterial activity of the oil was revealed with better susceptibility of Gram positive bacteria towards the oil. The MIC values ranged between 6–75 μg/ml. Besides, the oil demonstrated a moderate inhibitory effect on the three *Candida* species examined; with MIC values ranging between 48–95 μg/ml. Potent cytotoxic activities, especially against HeLa cell line; with LD_50_ of 32 μg/ml, BJAB cell line; with LD_50_ of 57 μg/ml, and Caco-2 cell line; with LD_50_ of 61 μg/ml, were observed.

**Conclusion:**

The results obtained indicate high potential of *Anthemis palestina* essential oil as bioactive oil, for nutraceutical and medical applications, possessing antioxidant, antimicrobial and antiproliferative activities.

## Background

The genus *Anthemis* is one of the most important genera of the Asteraceae family and comprises of approximately 210 species [1]. The geographic distribution of *Anthemis* extends across Europe, Southwestern Asia, Northern and Northeastern Africa, Southern Arabia, and tropical East Africa [2, 3]. Since the Roman times, the species of this genus have been commonly used in traditional medicine therapies [4, 5]. In the Mediterranean region, anti-inflammatory, antioxidant, antibacterial, and antispasmodic remedies are the most common traditional applications of the genus species.

During the last two decades, chemical composition of various extracts of *Anthemis* species was thoroughly investigated. Basically, flavonoids, polyphenolics and terpenes were reported as the main constituents of the plant [6, 7]. Recently, numerous *Anthemis* species have shown potential antimicrobial activity that could be correlated to their pehnolics and flavanoids composition [7, 8]. Additionally, digestive, antispasmodic and anti-Helicobacter pylori activities were all linked to *Anthemis* species in several reports [9–11]. On the other hand, the essential oils from different *Anthemis* species are frequently encountered as preservatives and flavoring agents in pharmaceuticals and cosmetic products [12–16].

*Anthemis palestina* Reut. ex Boiss., is distributed in the Middle and the Northern mountainous regions of Jordan, where it is known as Palestine chamomile due to resemblance to Roman and German chamomiles; the plant is an annual herb with flowering period between March-June [17]. Despite the extensive use of *A. palestina* in Folk medicine in Jordan, there have been only limited attempts to investigate the chemical or the biological properties of this plant in relation to its medicinal uses. Nonetheless, chemical composition of the essential oil isolated from flowers of *A. palestina* was recently reported by Tawaha *et al.* [18]. In the present study, in an effort to characterize the biological activities of *A. palestina,* we report here the antioxidant, antimicrobial, and antiproliferative activities associated with the essential oil isolated from the air-dried flowers of the plant. To our knowledge, thoroughly detailed studies on different aspects of the biological activity of *A. palestina* have not been reported yet.

## Methods

### Plant materials

The dried flowers of *Anthemis palestina* Reut. ex Boiss. were collected from Houfa region, Irbid (Northern Jordan), during its flowering phase (March to May), in Mid-April, 2012. The taxonomic identity of the collected plant was confirmed by Eng. Mohammad Al-Gharaibeh, a botanist, Department of Natural Resources and Environment, Faculty of Agriculture, Jordan University of Science and Technology (JUST), Irbid, Jordan. A voucher specimen (No. KT-AP-JOR-2012) has been deposited at the herbarium of the same institute.

### Essential oil isolation

The air-dried flowering parts (whole flowers) of the collected plant were ground to about 0.5 mm particle size (30–35 mesh). To obtain the essential oil, 500 g of the ground plant material were accurately weighed and subjected to hydrodistillation using Clevenger-type apparatus for 4 hours. The hydrodistilled oil (yield ~ 0.7%) was dried over anhydrous sodium sulphate and reserved at 4°C in a sealed brown vial until required.

### Essential oil composition analysis

Oil composition analysis was achieved via GC-FID and GC-MS analysis as reported in details in our previous In-Press work by Tawaha *et al*. [18]. Briefly, samples of the oil were injected to both system and analyzed under linear temperature programming. Compounds identification was performed by matching their spectra with the data bank mass spectra (WILEY, NIST and ADAMS-2007 libraries) and also by comparing their calculated arithmetic indices with reported values in the literature including the Adam’s library [18]. Identification of the main components was further confirmed by co-chromatography of their authentic standards under the same chromatographic conditions of samples analysis [18].

### Antioxidant activity

#### DPPH free radical scavenging activity

The free-radical scavenging activity of the hydrodistilled oil was measured as a decrease in the absorbance of methanol solution of 2,2-diphenyl-1-picrylhydrazil (DPPH). A stock solution of DPPH (0.1 mM) was prepared in methanol and different concentrations of the essential oil were added (5–250 μg/ml). After incubation at room temperature for 30 min, the pale pink color developed was measured spectrophotometerically at 517 nm and compared with the standard (5–250 μg/ml ascorbic acid) [19]. Free radical scavenging activity was expressed as the percentage inhibition calculated using the following formula: % Anti-radical activity = (Control Absorbance- Sample Absorbance) × 100/Control Absorbance.

#### Hydroxyl radical scavenging activity

The oil (0.2 ml) at different concentrations (5–250 μg/ml), was added to 1 ml of iron-EDTA solution (0.13% ferrous ammonium sulphate and 0.26% EDTA), 0.5 ml of 0.018% EDTA, 1 ml of DMSO (0.85% v/v in 0.1 M phosphate buffer, pH 7.4) and 0.5 ml of 0.22% ascorbic acid were added to each tube. The tubes were capped tightly and heated in a water bath at 80-90°C for 15 min. The reaction was terminated by adding 1 ml of ice-cold trichloroacetic acid (TCA) (17.5% w/v). Afterward, 3 ml of Nash reagent (75.0 g of ammonium acetate, 3 ml of glacial acetic acid and 2 ml of acetyl acetone were mixed and distilled water was added to make up total volume of 1 L) was added to each tube, then left at room temperature for 15 min for color development. The intensity of the yellow color formed was measured at 412 nm against blank [20]. Percentage inhibition was determined by comparing the results of the test and the standard compound (ascorbic acid) by using the formula: % inhibition activity = (Control Absorbance- Sample Absorbance) × 100/Control Absorbance.

#### Ferric-reducing antioxidant power (FRAP) assay

The reducing power of the oil was determined by the Ferric-Reducing Antioxidant Power (FRAP) assay described by Yen and Chen [21]. One ml of different concentrations of the oil (5–250 μg/ml) was mixed with 2.5 ml of potassium phosphate buffer (0.2 M, pH 6.6) and 2.5 ml of potassium ferricyanide (1 g/100 ml). The mixture was incubated at 50°C for 20 minutes. TCA (10%, 2.5 ml) was added to the mixture to terminate the reaction. Finally, 2.5 ml of the supernatant solution was mixed with 2.5 ml distilled water and 0.5 ml ferric chloride. The procedure was repeated in triplicate and allowed to stand for 30 min before measuring the absorbance at 700 nm. Ascorbic acid was used as a positive control. The percentage of antioxidant activity in FRAP assay of the samples was calculated according to the formula:


Where,

A_0_ = Absorbance of the control (potassium phosphate buffer + FRAP reagent)

A_1_ = Absorbance of sample

### Antimicrobial activity

#### Microorganisms

Six bacterial and three fungal species were obtained from the Microbial Culture Collection Center of Medicine School at The University of Jordan, Jordan. The species are: *Bacillus subtilis* ATCC 11562, *Staphylococcus aureus* ATCC 6538, *Staphylococcus epidermidis* ATCC 12228, *Escherichia coli* ATCC 29425, *Pseudomonas aeuriginosa* ATCC 11921, *Xanthomonas vesicatoria* ATCC 11633, *Candida albicans* ATCC 10231*, Candida glabrata* ATCC 1615, and *Candida krusei* ATCC 6258.

#### Determination of minimum inhibitory concentration (MIC)

The MICs of the oil against the microorganisms under investigation were assessed according to the broth microdilution method, as previously reported [22], with minor modifications. Briefly, 0.3 ml of the oil was dissolved in DMSO and serial diluted with the medium to the desired concentrations. MIC tests were carried out in 96 flat bottom microtiter plates (TPP, Switzerland). Each test well was filled with 100 μl nutrient broth. A sample (100 μl) of the stock solution was added to the first test well and mixed. A series of dilutions was then prepared across the plate. A 10 μl aliquot of the microorganism was used to inoculate each microtiter plate well to achieve a final inoculum size of 1 × 10^5^ CFU/ml. Bacteria were grown in Mueller-Hinton broth (MHB; Oxoid, Basingstoke, UK) whereas *Candida* were grown in Yeast Peptone Dextrose (YPD) broth (BD Difco™). Positive controls, Norfloxacin for bacteria and Amphotericin B for fungi, and a negative control of the vehicle (DMSO), were prepared under the same experimental conditions and employed in triplicates.

Bacterial plates were incubated for 24 h at 37°C, whereas *Candida* plates were incubated for 48 h at 33°C, with shaking. After completion of the incubation period, optical densities were measured at 600 nm (OD_600_) using a Microplate Reader (Palo Alto, CA, USA). The minimum inhibitory concentration (MIC) value was defined as the lowest concentration that inhibited visible microbial growth of the tested microorganism. MIC determination was carried out in triplicates (in same 96-well plate) and repeated three times for each microorganism.

### Antiproliferative activity

#### Cell culture

Cell lines were cultured in high glucose Dulbecco’s Modified Eagle Medium (DMEM) (Invitrogen, USA) containing 10% heat inactivated fetal bovine serum (HI-FBS) (Invitrogen), 2 mmol/l *L*-glutamine, 50 U/ml penicillin and 50 μg/ml streptomycin. Cell lines were maintained at 37°C in a 5% CO_2_ atmosphere with 95% humidity. The cells were passaged weekly, and the culture medium was changed twice a week. The optimal plating densities were determined according to the cells growth profiles,

#### MTT assay

Cytotoxicity was determined using the 3-(4,5-dimethylthiazol-2-yl)-2,5-diphenyltetrazoliumbromide (MTT, Sigma) assay, as previously described [23]. The concentration that inhibits the proliferation of oil-treated cells by 50% relative to the control (untreated cells) was used to evaluate the cytotoxic activity of the essential oil. To ensure exponential growth throughout the experimental period, as well as a linear relationship between absorbance at 570 nm and cell number when analyzed by the MTT assay, 2 × 10^4^ cells per well were seeded of each cell line. Treated cells were incubated in a 37°C incubator with 5% CO_2_ for 48 h. As a positive control, vincristine sulphate (Sigma) was used at concentrations of 50 and 100 nM. In addition, a negative control; control wells without essential oils treatment, was employed and prepared under the same experimental conditions. All treatments were carried out in triplicates (in same 96-well plate) and repeated two times for each cell line.

### Statistical analysis

All results were expressed as the mean ± standard deviation (SD). Data were analyzed with Statistical Package for Social Sciences program (SPSS) database for Windows, version 17 (SAS Institute, Cary, NC). The t-test was used to test for statistical significance with a p-value <0.05 deemed as significant.

## Results and discussion

The simultaneous use of mass spectral and retention (Kovat’s) index matching allowed for the unequivocal identification of more than 97% of the components of the oil, obtained from flowers, as previously determined by the GC and GC-MS analyses [18]. The oil yield was 0.7% (v/w dried material). The analyses permitted the identification of about 109 compounds in the oil of *A. palestina*. The oil was characterized by dominant levels of sesquiterpenes (71.4%, hydrocarbons = 26.6% and oxygenated = 44.8%) and a moderate level of monoterpenes (19.4%), mainly oxygenated (18.5%). Spathulenol (9.8%) was the principal oil component, where, germacrene D (8.9%), caryophyllene oxide (6.8%), 3-thujanol acetate (3.7%), E-β-farnesene (3.5%), bornyl andelate (3.2%), 1,8-cineole (2.9%), salvial-4(14)en-1-one (2.5%), α-cadinol (2.4%), β-caryophyllene (2.3%) and some other components were the major oil constituents.

**Figure 1:**
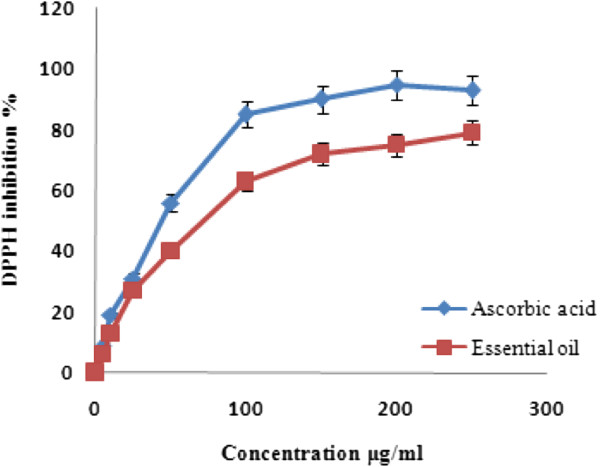
**Free radical scavenging activity of** ***Anthemis palestina*** **essential oil at different concentrations.** Values are mean ± SD of three experiments.

An antioxidant is defined as a substance that considerably suspends or inhibits an oxidation process [24]. The antioxidant activity is commonly determined via measuring the inhibition rate of an oxidation processes in the presence of an antioxidant [24]. Antioxidant efficiency is often linked to the antioxidant ability to scavenge stable free radicals. DPPH is extensively used to determine the antiradical activity of a given compound or extract. Figure 1 demonstrates the free radical scavenging activity of *A. palestina* essential oil relative to a standard antioxidant, such as ascorbic acid. As illustrated, the tested oil exhibits a dose-dependent scavenging activity against DPPH activity. In fact, the oil yielded percentage scavenging activities of 13%, 27%, 40% and 63% at concentrations of 10, 25, 50 and 100 μg/ml, respectively (Figure 1). Apparently, the oil of *A. palestina* possesses a moderate radical scavenging activity relative to the strong antioxidant ascorbic acid (p-value <0.05), which demonstrated 85% radical scavenging activity at 100 μg/ml (Figure 1).

**Figure 2:**
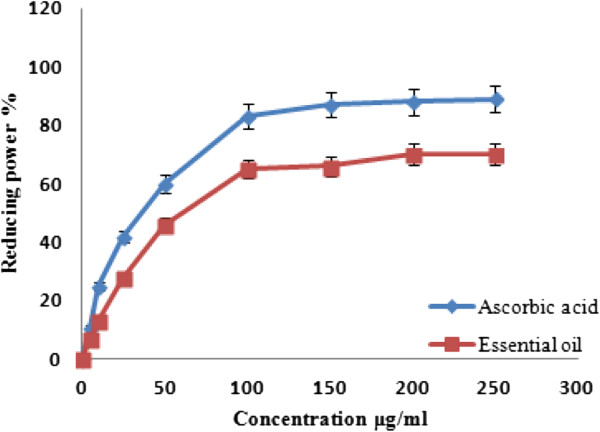
**Reducing power activity** ***Anthemis palestina*** **essential oil at different concentrations.** Values are mean ± SD of three experiments.

Furthermore, the ability of the *A. palestina* oil to reduce iron (III) to iron (II) was determined and compared to ascorbic acid, as shown in Figure 2. The results illustrate that the essential oil was able to reduce iron in a dose-dependent manner with a maximum reducing power of 65% at 100 μg/ml, whereas ascorbic acid resulted in 89% reduction efficiency of the sample (Figure 2), at the same concentration (p-value <0.05).

**Figure 3:**
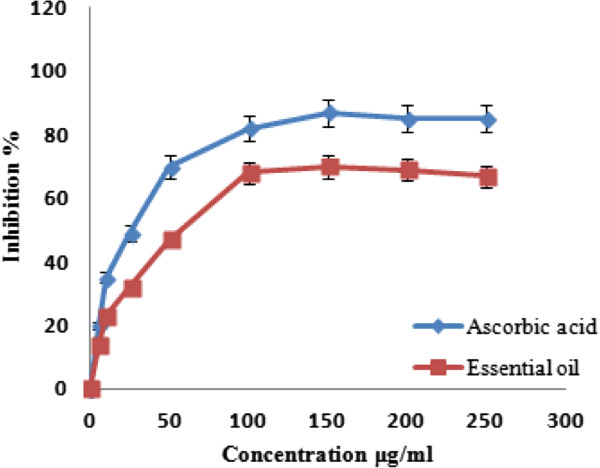
**Hydroxyl radical scavenging activity of** ***Anthemis palestina*** **essential oil at different concentrations.** Values are mean ± SD of three experiments.

In addition, the hydroxyl (OH^.^) reactive oxygen species (ROS) is one of the most reactive and physiologically harmful free radicals. In this study, the concentration of the essential oil needed for scavenging 50% of the hydroxyl activity was found at 56 μg/ml, whereas that for the standard antioxidant, ascorbic acid, was at 45 μg/ml (Figure 3). Seemingly, the scavenging activity possessed by the essential oil is comparable with the strong antioxidant ascorbic acid (p-value <0.05).

Free radicals are the most common initiators of oxidative reactions that may result in numerous deleterious effects [24]. Natural antioxidants can scavenge and react with free radicals, and hence terminate the free radical reaction. Recently, herbal medicines containing free radical scavengers are drawing attention of the pharmaceutical research for their importance in preventing and treating several diseases and disorders [25]. Generally, essential oils were shown to protect against oxidative stress by contributing to the total antioxidant defense system of the human body [26]. Recently, a number of essential oils isolated from several medicinal and aromatic plants were shown to possess considerable antioxidant potential and, consequently, protect against some cardiovascular and degenerative diseases [26–28]. Our findings demonstrate that the essential oil of *A. palestina* has a substantial antioxidant potential that may be attributed to its chemical composition.

Furthermore, the oil from *A. palestina* was assayed for its *in vitro* antibacterial activity, using the microdilution method, towards six different bacterial species, representing both Gram positive and Gram negative bacterial (Table 1). Literature review revealed that there are no previous reports about the antibacterial activity of *A. palestina*. Our results indicated that the essential oil exhibited a broad-spectrum antibacterial activity. Interestingly, the results demonstrated that Gram-positive bacteria were more susceptible to the oil treatment than Gram-negative bacteria. Particularly, *Staphylococcus epidermidis* (MIC 6 ± 1 μg/ml) was the most susceptible Gram positive bacteria while *Escherichia coli* (MIC 53 ± 4 μg/ml) was the most affected Gram negative bacteria (Table 1).

**Table 1:** **The MIC values (μg/ml) of** ***Anthemis palestina*** **essential oil**

Microorganism	MIC (μg/ml)
***Gram positive bacteria***	
*Bacillus subtilis*	
*ATCC 11562*	25 ± 6
*Staphylococcus aureus*	
*ATCC 6538*	10 ± 2
*Staphylococcus epidermidis*	
*ATCC 12228*	6 ± 1
***Gram negative bacteria***	
*Escherichia coli*	
*ATCC 29425*	53 ± 4
*Pseudomonas aeuriginosa*	
*ATCC 11921*	75 ± 8
*Xanthomonas vesicatoria*	
*ATCC 11633*	73 ± 6
***Fungi***	
*Candida albicans*	
*ATCC 10231*	48 ± 5
*Candida glabrata*	
*ATCC 1615*	86 ± 4
*Candida krusei*	
*ATCC 6258*	95 ± 3

Values are expressed as mean ± SD of three experiments.

Notably, the essential oil of *A. palestina* exhibited moderate antifungal activity against *Candida albicans*; with MIC value of 48 ± 5 μg/ml, *Candida glabrata*; with MIC value of 86 ± 4 μg/ml, and *Candida krusei*; with MIC value of 95 ± 3 μg/ml (Table 1). The increased incidence of systemic candidiasis infections, caused by pathogenic yeasts in critically ill patients [29], as well as the emergence of resistant *Candida* species to several antifungal drugs, necessitates a comprehensive search for newer drugs to treat candidiasis. Chemical diversity of medicinal plants may provide unlimited prospects for new therapeutic reagents discovery and development. Our findings indicate that the essential oil of *A. palestina* may have a potential for the development of new antifungal, as well as antibacterial agents, and demonstrate the importance of such medicinal plant in pharmaceutical production and industry.

**Figure 4:**
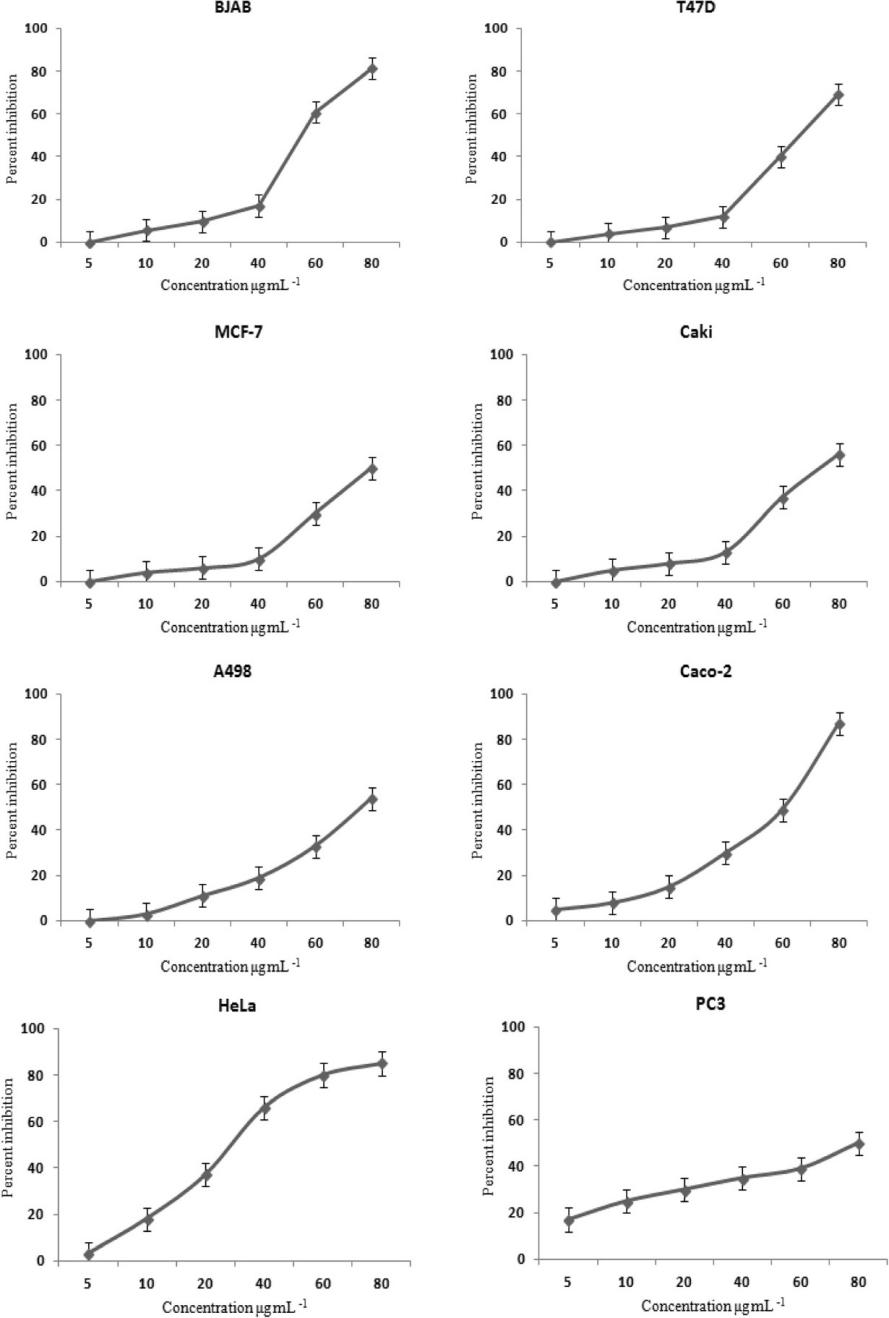
**Cytotoxic activities of** ***Anthemis palestina*** **essential oil against eight human cancer cell lines.** Exposure time 48 h. Values are expressed as mean ± SD of three experiments.

On the other hand, the plant hydrodistilled oil was further evaluated for its *in vitro* cytotoxic properties on eight human cancer cell lines, including human breast adenocarcinoma MCF-7 cell line, the human ductal breast epithelial tumor T47D cell line, the EBV-negative Burkitt’s lymphoma BJAB cell line, the human colon adenocarcinoma Caco-2 cell line, the human epithelial carcinoma HeLa cell line, the human prostate adenocarcinoma PC-3 cell line, the human clear cell renal cell carcinoma Caki cell line, and the human kidney carcinoma A498 cell line. It is known that different cell lines might demonstrate variable sensitivities to different plant extracts and, therefore, the use of more than one cell line appears compulsory for the comprehensive anti-cancer activity screening. Cytotoxic effects of the oil on the growth of the studied cell lines are presented in Figure 4, which shows the percentage of growth inhibition versus the concentration of the oil. The concentration of the oil at which growth was inhibited by 50% (LD_50_) was calculated based on the dose-dependent curves (Table 2). According to the demonstrated results, the oil exhibited potent *in vitro* cytotoxic properties on HeLa (LD_50_ 32 μg/ml), Caco-2 (LD_50_ 61 μg/ml), and BJAB cell lines (LD_50_ 57 μg/ml). The variation in cytotoxic effects observed in different cell types is likely due to the versatility of the oil composition and the different ways the oil components may interact with different cell types [30]. Interestingly, the presence of 1,8-cineole, as one of the main oil constituents, supports its cytotoxic activity as suggested previously [31, 32].

**Table 2:** **The LD**_**50**_ **values (μg/ml) of** ***Anthemis palestina*** **essential oil on eight human cancer cell lines**

Cell line	LD _50_(μg/ml)
*EBV-negative Burkitt’s lymphoma*	
***BJAB***	57 ± 2
*Human ductal breast epithelial tumor*	
***T47D***	71 ± 2
*Human breast adenocarcinoma*	
***MCF-7***	79 ± 4
*Human clear cell renal cell carcinoma*	
***Caki***	78 ± 3
*Human kidney carcinoma*	
***A498***	76 ± 2
*Human colon adenocarcinoma*	
***Caco-2***	61 ± 3
*Human epithelial carcinoma*	
***HeLa***	32 ± 2
*Human prostate adenocarcinoma*	
***PC-3***	80 ± 6

Values are expressed as mean ± SD of three experiments.

Furthermore, literature reports on the composition-activity correlations were found in support with the findings of the present study. For example, in literature reviews concerning essential oils as active antimicrobials [33–35], it was reported that most of the antimicrobial activity of the essential oils is attributed to the oxygenated monoterpenes [35]. In particular, it was evident that monocyclic monoterpenes, such as 1,8-cineole and terpinen-4-ol, exhibit high antimicrobial activity against a wide range of Gram-positive and Gram-negative bacteria [33–35]. Additionally, sesquiterpenes caryophyllene, germacrene D, caryophyllene oxide, and spathulenol were also reported among the most potent antimicrobial agents frequently occurring in essential oils [33–35]. Nonetheless, since essential oils consist of multiple components, their antimicrobial activities cannot be assigned to one particular component. In most cases their activity is a result of additive, synergistic or antagonistic effects of the individual constituents. Therefore, the principle oil component is generally assumed to possess the strongest activity that can be influenced by the presence of other oil components.

Similarly, cytotoxic activities of essential oils from various aromatic plants were attributed to specific components of the oils [36, 37]. Some of the main components of *A. palestina* oil have previously been tested for cytotoxic properties. For example, β-caryophyllene was reported to be specifically cytotoxic to MCF-7, MDA-MB-468 and UACC-257 cancer cell lines [38].

On the other hand, antioxidant activity is one of the most intensively studied subjects in essential oil research [39, 40]. The oxygenated constituents, specifically phenolic monoterpenic alcohols (e.g. thymol and carvacrol), as well as phenylpropanoids (e.g. eugenol), are the most powerful antioxidant volatiles [41]. Nevertheless, some non-phenolic terpenoids were also reported to be of significant antioxidant capacities [41]. Additionally, Many other components of essential oils were proposed to contribute to the antioxidant activity of essential oils [42]; including α-terpinene, β-terpinene, β-terpinolene, 1,8-cineole, menthone, isomenthone, and citronellal [42]. Accordingly, the high percentage of oxygenated terpenoids and the particular presence of some alcohols, as 1,8-cineole and terpinen-4-ol, in the oil of *A. palestina* would rationalize its relatively high antioxidant activity.

## Conclusions

In conclusion, our results revealed that the essential oil of *Anthemis palestina* exhibits considerable antioxidant activity and can be used as alternative medicine to prevent or treat oxidative stress. Furthermore, the examined oil demonstrates moderate antibacterial and antifungal activities elucidated by the growth inhibitory response against clinically problematic microorganisms. The discrepancy observed in the cytotoxicity activities of the oil against different cancer cell lines suggests a unique antiproliferative mechanism through which the oil exhibits its anticancer effects. Further research into the molecular mechanisms, as well as isolation and characterization of the chemical constituents responsible for the activity would be necessary to identify the active components with potential for clinical use.
